# Paclitaxel Protects against Isoproterenol-Induced Damage in Rat Myocardium: Its Heme-Oxygenase Mediated Role in Cardiovascular Research

**DOI:** 10.3390/antiox12051129

**Published:** 2023-05-20

**Authors:** Danica Matusovits, Zsolt Murlasits, Krisztina Kupai, Zoltán Baráth, Hsu Lin Kang, Péter Osváth, Miklós Szűcs, Dániel Priksz, Béla Juhász, Zsolt Radák, Tamás Várkonyi, Imre Pavo, Anikó Pósa

**Affiliations:** 1 Department of Prosthodontics, Faculty of Dentistry, University of Szeged, 6703 Szeged, Hungary; matusovits.danica@stoma.szote.u-szeged.hu; 2 Institute of Sport Science and Physical Education University of Pécs, 7601 Pécs, Hungary; 3 Department of Internal Medicine, Albert Szent-Györgyi Medical School, University of Szeged, 6703 Szeged, Hungary; kupai.krisztina@med.u-szeged.hu (K.K.); varkony.tamas@med.u-szeged.hu (T.V.); pavo_imre@lilly.com (I.P.); 4 Department of Oral Biology and Experimental Dental Research, Faculty of Dentistry, University of Szeged, 6703 Szeged, Hungary; barath.zoltan@stoma.szote.u-szeged.hu (Z.B.); hsu.lin-kang@o365.u-szeged.hu (H.L.K.); aniko.posa@icloud.com (A.P.); 5 Department of Urology, University of Debrecen, 4006 Debrecen, Hungary; dr.peter.osvath@kenezy.unideb.hu (P.O.); dr.szucs.miklos@kenezy.unideb.hu (M.S.); 6 Department of Pharmacology and Pharmacotherapy, Faculty of Medicine, University of Debrecen, 4006 Debrecen, Hungary; priksz.daniel@pharm.unideb.hu (D.P.); juhasz.bela@med.unideb.hu (B.J.); 7 Institute for Sports and Health Sciences, Hungarian University of Sports Science, 1051 Budapest, Hungary; radak@tf.hu

**Keywords:** myocardial injury, inflammation, paclitaxel, isoproterenol, cardioprotection

## Abstract

(1) Background: In cardiovascular applications, paclitaxel inhibits smooth muscle cell proliferation and migration and significantly reduces the occurrence of restenosis and target lesion revascularization. However, the cellular effects of paclitaxel in the myocardium are not well understood; (2) Methods: Wistar rats were divided into four groups: control (CTRL), isoproterenol (ISO) treated (1 mg/kg) and two groups treated with paclitaxel (PAC), which was administrated (10 mg/kg/day) for 5 days by gavage/per os alone or in combination (ISO + PAC) 3 weeks after ISO treatment. Ventricular tissue was harvested 24 h later for measurements of heme oxygenase (HO-1), reduced glutathione (GSH), oxidized glutathione (GSSG), superoxide dismutase (SOD), NF-κB, TNF-α and myeloperoxidase (MPO); (3) Results: HO-1 protein concentration, HO-1 activity, SOD protein concentration and total glutathione significantly decreased in response to ISO treatment. When PAC was administered in conjunction with ISO, HO-1, SOD concentration and total glutathione were not different from control levels. MPO activity, NF-κB concentration and TNF-α protein concentration were significantly increased in the ISO-only group, while the levels of these molecules were restored when PAC was co-administered; (4) Conclusions: Oral administration of PAC can maintain the expression of important antioxidants, anti-inflammatory molecules, HO-1, SOD and GSH, and suppress the production of TNF-α, MPO and NF-κB, which are involved in myocardial damage. The principal component of this cellular defense seems to be the expression of HO-1.

## 1. Introduction

Paclitaxel is a highly cytotoxic compound isolated from the bark of the Pacific yew tree. Due to its antiproliferative properties, it has been used for decades in oncology as a standard therapy for breast cancer, lung cancer and ovarian cancer, among others. In cardiovascular applications, paclitaxel was first used in coronary vessels, and it is now a common coating-substance of drug-eluting stents (DES) and drug-coated balloons (DCB). In this context, the drug inhibits smooth muscle cell proliferation and migration and significantly reduces the occurrence of restenosis and target lesion revascularization with few side effects [1,2,3,4,5,6]. Paclitaxel also plays a role in the regulation of inflammation. Mechanistically, these functions are carried out by the irreversible stabilization of the microtubules, resulting in dysfunctional structures that disrupt crucial cellular processes, such as cellular signaling and the maintenance of organelle organization [7,8].

Today, paclitaxel-coated stents and balloons are commonly used in clinical application to reduce the high rate of post-interventional restenosis seen with standard procedures [5,9]. A recent meta-analysis, however, raised questions regarding the long-term safety of paclitaxel when it reported that compared to alternative therapies, drug-coated devices increased the risk of mortality at two and five years in patients undergoing peripheral artery procedures [10]. However, the authors were not able to explain the potential mechanisms of the late mortality. This was a shocking finding, because paclitaxel has been used for a long time as an antiproliferative agent in cancer therapy without any indication of related mortality. Moreover, in endovascular procedures, the coating dose of paclitaxel is typically less than 10% of what is administered in a single dose in cancer treatment [1]. The work also generated a lot of criticism, because it failed to account for patient withdrawal and loss to follow up, and also lacked patient-level analyses. Subsequent clinical trials also disagreed with the conclusion of this meta-analysis [11,12]. First, Zeller et al. [11] indicated that paclitaxel administration was not associated with increased risk of amputation or all-cause mortality at five-year follow up after treatment of infrapopliteal arterial disease. In addition, a German study analyzed health insurance data and found no difference in mortality rate for over 11 years between paclitaxel-coated balloons/paclitaxel-eluting stents and other devices without drugs [12].

Elucidating the molecular mechanisms of paclitaxel action in vascular and cardiac cells may provide clues regarding its safety, efficacy and specific applications in cardiovascular medicine. However, beneficial antioxidant functions as well as damaging effects were demonstrated for this drug by previous research studies in the area. Paclitaxel reduced the severity of ischemic ventricular arrhythmias and the extent of ischemia-reperfusion injury when administered in 0.1 μM and 1 μM concentrations in isolated rat hearts, with the higher dose having a larger effect in both cases [13]. Subsequently, the same research group demonstrated that taxol in 0.1 μM, 0.3 μM and 1 μM doses reduced the production of reactive oxygen species (ROS) and induced heme oxygenase (HO-1) expression, which can potentially explain the previously reported protective effects [14]. Based on these works, the authors speculated that microtubular stabilization may have contributed to the observed effects via reduced intracellular Ca^2+^ overload. In contrast to these results, others did not detect any cardioprotective effect of paclitaxel despite the apparent microtubule stabilization [8]. This work also reported increased cytosolic Ca^2+^ levels, which may explain the lack of cardioprotection in this model. In another investigation, paclitaxel induced mitochondrial permeability transition pore (mPTP) opening in cardiac myocytes, which was explained by the elevated ROS levels in the cells [15]. Moreover, long-term paclitaxel administration led to increased TNF-α protein level and apoptosis in mice hearts [16]. The different (1–10 μM) doses and protocols used in these studies might account for the discrepancies, as higher drug exposures seem excessive and may even produce toxic effects. The dose-dependency was also demonstrated by Kang et al. [17] as low and high paclitaxel concentrations were less effective in decreasing the proliferation of smooth muscle cell-derived foam cells. Interestingly, high doses (≥1 μM) increased the inflammatory interleukin 6 (IL-6) and decreased the anti-inflammatory interleukin 35 (IL-35) cytokine levels. 

Although a plethora of research exists regarding paclitaxel in oncological and vascular applications, the lack of sufficient data makes it difficult to draw conclusions about the cellular effects of paclitaxel in the myocardium. Therefore, in this investigation we aimed to analyze the potential cardioprotective properties of paclitaxel in the myocardium of rats subjected to isoproterenol-induced heart damage. Isoproterenol is a non-selective α-adrenergic agonist which is widely used to induce myocardial damage via free radical production [18]. We hypothesized that paclitaxel administration would protect the heart by suppressing oxidative and inflammatory processes, such as the TNF-α and NF-κB canonical proinflammatory signaling pathway, and by stimulating the expression of protective molecules, such as HO-1 and Superoxide Dismutase (SOD).

## 2. Materials and Methods

### 2.1. Animals

In the present study, adult male Wistar rats (BRC, Szeged, Hungary) were kept and housed in cages under constant temperature (20–22 °C) and humidity (40–50%) for 10 days with a 12/12 h light/dark cycle before the experiments. The rats received food and water ad libitum and weighed 150–200 g. The body weight was measured at baseline and at the end of this trial as well. During our experiment, we kept the number of rats at the minimum necessary to complete the experiments. The sample size was determined based on similar previous experiments and pilot work in our laboratory and by resource equation calculation. All protocols were in compliance with the Directive of the European Parliament (2010/63EU) and were authorized by the European Community guidelines (XXXIX./3546/2022).

### 2.2. Experimental Protocol

**Figure 1:**
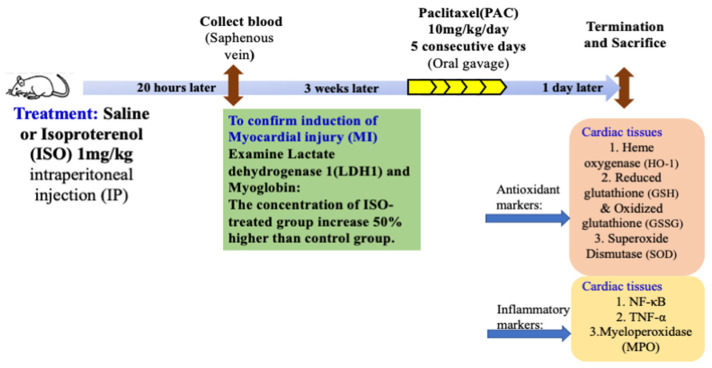
Experimental Timeline and Protocols.

Wistar rats were divided into 4 groups after acclimatization to the experimental conditions. Control rats (CTRL) were intraperitoneally (IP) injected with saline or isoproterenol (ISO) 1 mg/kg. Blood samples were collected from the saphenous vein 20 h later to determine LDH-1 and myoglobin isoform levels in order to verify myocardial injury (MI) after ISO treatment. Three weeks after ISO treatment, paclitaxel (PAC) was administrated (10 mg/kg/day) for 5 days by gavage/per os [19] both to the control rats without ISO damage (PAC) and to the rats previously treated with ISO (ISO + PAC). All rats were sacrificed 24 h after the oral gavage and the hearts were perfused to wash out the remaining blood. The left ventricles were then harvested for biological measurements: heme oxygenase (HO-1), reduced glutathione (GSH), oxidized glutathione (GSSG), superoxide dismutase (SOD), NF-κB, TNF-α and myeloperoxidase (MPO). The left ventricular samples were powdered under liquid nitrogen and stored at −80 °C until the analyses (Figure 1).

### 2.3. Determination of Cardiac HO-1, NF-kB and TNF-α Concentrations

Approximately 40 mg of the powdered ventricular sample was homogenized in an ice-cold phosphate buffer (pH 7.4) for 20 s and supernatants were obtained after centrifugation at 4 °C (20 min, 2500 rpm) for further ELISA and protein analyses. An amount of 40 μL of supernatants of cardiac tissue or 50 μL of standard solution was added into each well, including a monoclonal antibody based on the manufacturer’s instructions (Gen Asia Biotech Co., Ltd., Shanghai, China). Next, 10 μL of the secondary antibody labeled with biotin was added to each well, and then we added 50 μL Streptavidin-HRP to the supernatant and the standard; the following formed an immune complex with a biotin-labeled antibody. Subsequently, the plate was washed 5 times after 60 min incubation at 37 °C to deplete unbound enzymes. Substrate A and B were added (50 μL) into the wells and incubated for 10 min at 37 °C for the purpose of color development. At the final step, 50 μL of the stop solution was added into the wells and pipetted, resulting in a color change from blue to yellow. We collected data at 450 nm optical density in a Microplate reader (Bio-Rad, Hercules, CA, USA) (Table 1).

### 2.4. Measurement of Cardiac Total (GSH + GSSG) Level

We prepared buffer A (0.25 M sucrose, 20 mM Tris and 1 mM dithiothreitol (DTT)) and buffer B (0.1 M CaCl2, 0.25 M sucrose, 20 mM Tris and 1 mM DTT). Approximately 40 mg of the powdered ventricles were homogenized in buffer A initially and next in buffer B. The supernatant which we discarded was centrifuged for 30 min at 4 °C, and then the clear cytosolic fraction that remained was used for an enzyme assay. The reagents (20 μL 5,5-dithiobis-2-nitrobenzoic acid (DTNB), 140 μL nicotinamide adenine dinucleotide phosphate (NADPH), 10 μL glutathione reductase and 40 μL sample) were mixed together in a 96-well plate. The results were detected at 405 nm after 10 min by ONTB formation after the beginning of the reaction. GSH levels were expressed as nM per milligram protein (Table 1).

### 2.5. Examination of Superoxide Dismutase (SOD) in Cardiac Tissue

The powdered ventricular samples were homogenized according to the guidelines of the manufacturer for SOD activity, which was measured by a specific kit (Abcam, ab65354, Abcam, Cambridge, UK), and the increase in the absorbance of the water-soluble formazan dye was detected at 450 nm with a microplate reader to determine enzyme activity. The results were expressed as ng/mL (Table 1).

### 2.6. Determination of Cardiac MPO Activity

Approximately 40 mg of the powdered ventricles were homogenized in a phosphate buffer (pH 6.0) containing 0.5% hexadecyltrimethylammonium bromide. At first, the samples were put in liquid nitrogen and then in a water bath (37 °C), and this step was repeated 3 times. Next, samples were centrifuged (15,000× *g* for 15 min at 4 °C) and the supernatants were collected. On a 96-well plate, we added 280 μL (o-dianisidinediHCL along with 12 μL of sample or standard (diluted from peroxidase)) into the wells. After shaking for 59 s, MPO activity was detected at 490 nm and expressed as μU/mg protein (Table 1).

### 2.7. Determination of Cardiac HO Activity

We prepared an ice-cold buffer, containing 10.0 mM HEPES, 32.0 mM sucrose, 1.0 mM DTT, 0.10 mM EDTA, 10.0 g/mL trypsin inhibitor, 10.0 μg/mL leupeptin and 2.0 μg/mL aprotinin (pH 7.4) and then the cardiac tissue samples were homogenized in this buffer. The supernatant was discarded after centrifugation at 15,000× *g* for 20 min at 4 °C. The reaction mixture contained 150.0 μL supernatant, 2.0 mM glucose-6-phosphate, 0.14 U/mL glucose-6-phosphate dehydrogenase, 15.0 μM hemin, 120.0 μg/mL rat liver cytosol as a source of biliverdin reductase, 2.0 mM MgCl2_6H2O and 100.0 mMKH2PO4. The reaction was initiated with 100.0 μL reduced β-NADPH and then the reaction mix was incubated in the dark for 60 min at 37 °C. Bilirubin content was determined by optical density, which was measured at 465 and 530 nm, and the difference between the two densities was calculated. HO activity was defined as the amount of bilirubin (in nmol) produced per hour per milligram of protein (Table 1).

### 2.8. Statistical Analysis

All experiments were designed to generate groups of equal size using randomization. Group sizes represent the number of independent samples/animals, not technical replicates. Data are presented as the mean value of the group ± standard deviation (SD), unless otherwise stated. First, to compare endpoint parameters of treatment groups, normal distribution was estimated by the D’Agostino and Pearson normality test. Statistical analysis was then performed using one-way analysis of variance (ANOVA) followed by the Tukey post-test (if normality test was passed, and there was no significant variance inhomogeneity), or the Kruskal-Wallis test followed by Dunn’s post-test (when the previous normality test was not passed). Statistical analyses were carried out using the GraphPad Prism software for Windows, version 7.00 (GraphPad Software Inc., La Jolla, CA, USA). Probability values (*p*) less than 0.05 were considered significant and marked with asterisks on the graphs (*: *p* < 0.05; **: *p* < 0.01; ***: *p* < 0.001; ****: *p* < 0.0001).

## 3. Results

### 3.1. HO-1 Protein Expression

**Figure 2:**
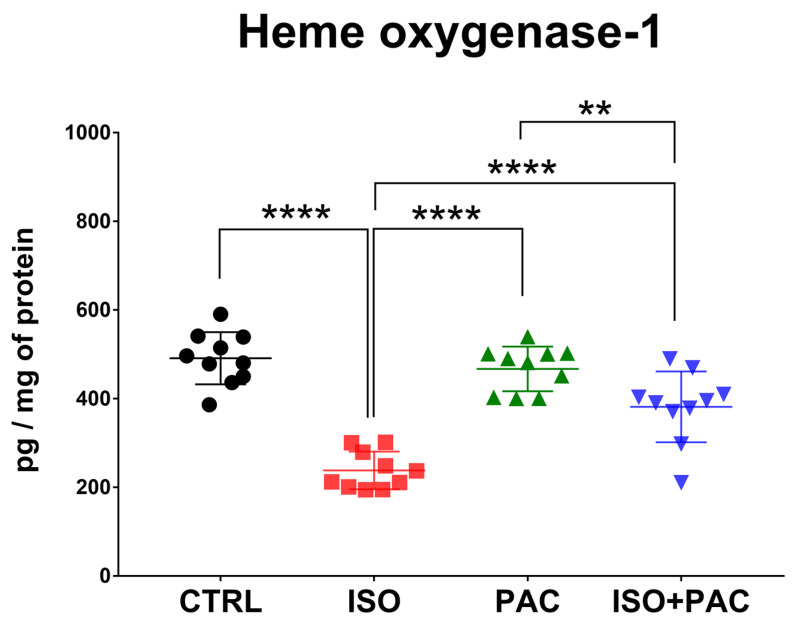
Heme oxygenase-1 protein concentration in CTRL-, ISO- and PAC-treated rat hearts. **: *p* < 0.01; ****: *p* < 0.0001.

HO-1 protein concentration was significantly decreased in response to isoproterenol treatment, while paclitaxel administration alone did not affect HO-1 protein expression in normal rat hearts. When paclitaxel was administered in conjunction with isoproterenol, HO-1 concentration was not different from control levels (Figure 2).

### 3.2. HO-1 Protein Activity

**Figure 3:**
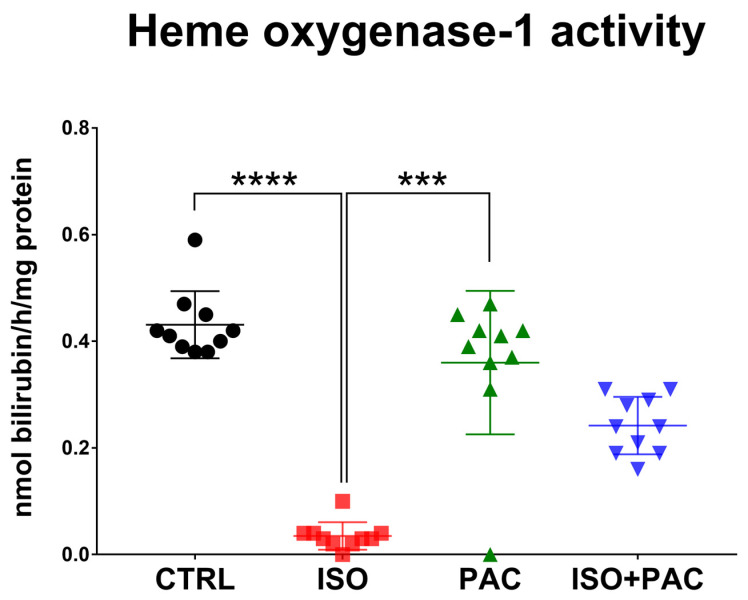
Heme oxygenase-1 activity in CTRL-, ISO- and PAC-treated rat hearts. ***: *p* < 0.001; ****: *p* < 0.0001.

HO-1 activity was also significantly decreased in response to isoproterenol treatment, while paclitaxel administration alone did not affect the activity of the enzyme in rat hearts. When paclitaxel was co-administered with isoproterenol, HO-1 activity was not statistically different from the suppressed activity by isoproterenol (Figure 3).

### 3.3. Superoxide Dismutase

**Figure 4:**
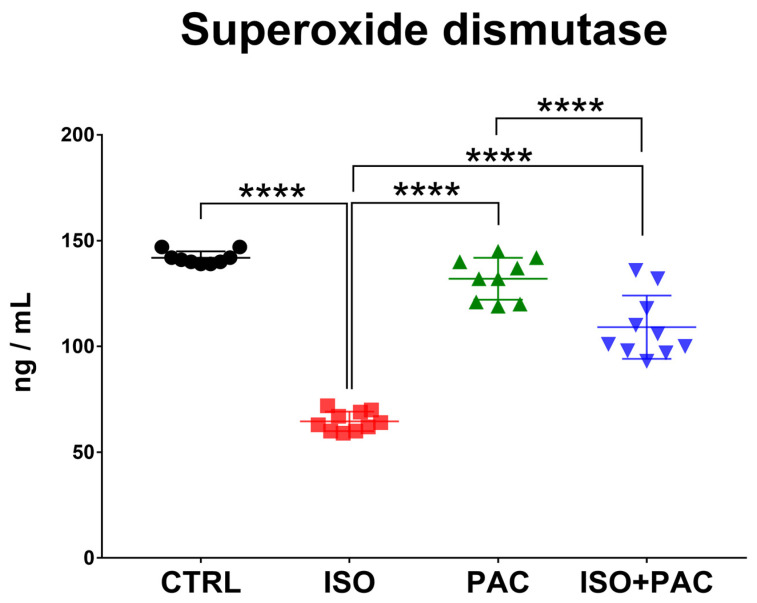
Superoxide dismutase concentrations in CTRL-, ISO- and PAC-treated rat hearts. ****: *p* < 0.0001.

SOD protein concentration was significantly decreased in the isoproterenol-only group, while paclitaxel administration alone did not affect protein expression in control hearts. When paclitaxel was administered in conjunction with isoproterenol, SOD concentration returned towards control levels with no statistically significant difference between these groups (Figure 4).

### 3.4. Total Glutathione

**Figure 5:**
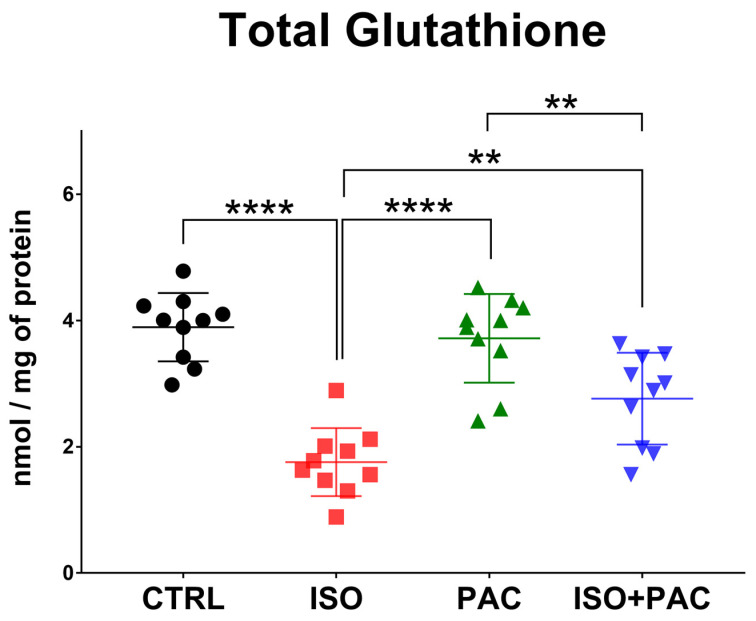
Total glutathione levels in CTRL-, ISO- and PAC-treated rat hearts. **: *p* < 0.01; ****: *p* < 0.0001.

Total glutathione decreased with isoproterenol administration, but remained at control levels when paclitaxel was used alone. Co-treatment with isoproterenol and paclitaxel significantly increased the total glutathione level compared to the isoproterenol-only group, without any difference from the control (Figure 5).

### 3.5. Myeloperoxidase

**Figure 6:**
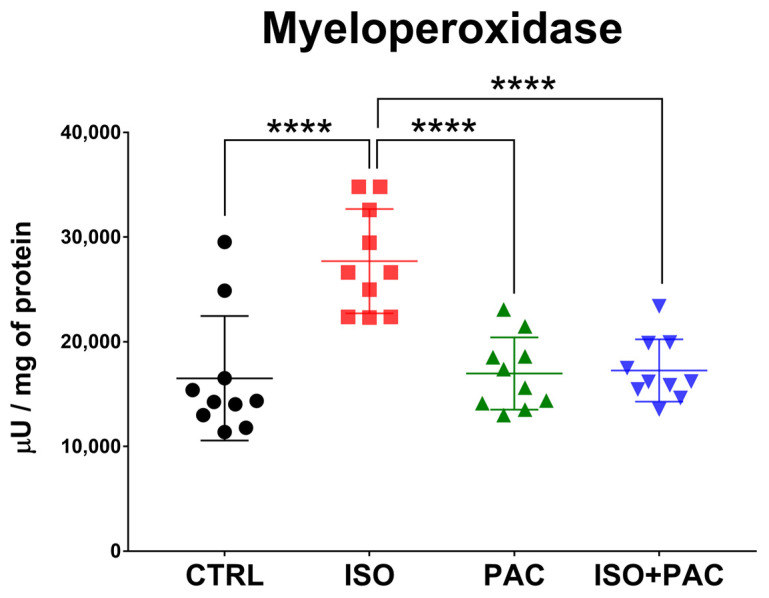
Myeloperoxidase activity in CTRL-, ISO- and PAC-treated rat hearts. ****: *p* < 0.0001.

MPO activity increased in the isoproterenol-treated group, which was restored to control levels when paclitaxel was co-administered. Paclitaxel alone had no effect on MPO activity (Figure 6).

### 3.6. NF-κB Protein Expression

**Figure 7:**
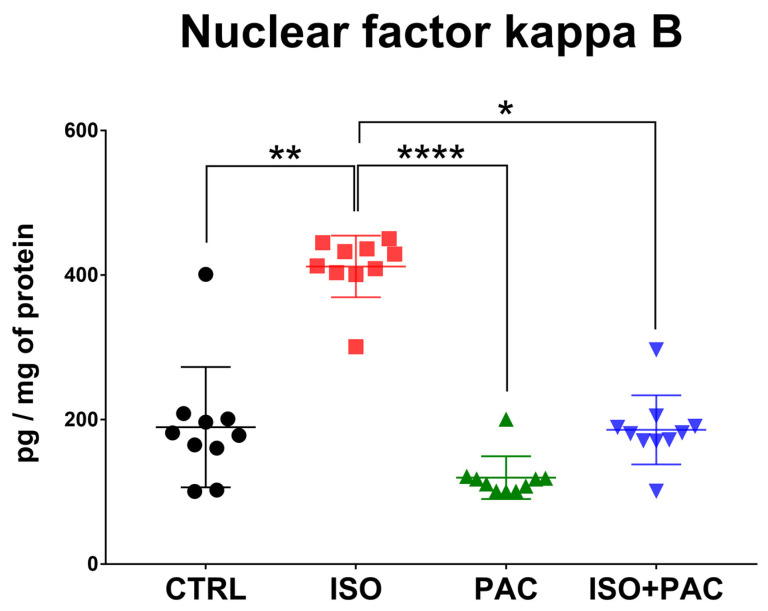
Nuclear factor-κB protein in CTRL-, ISO- and PAC-treated rat hearts. *: *p* < 0.05; **: *p* < 0.01; ****: *p* < 0.0001.

Isoproterenol significantly increased the NF-κB concentration, while paclitaxel alone had no effect on its expression. When paclitaxel was administered in conjunction with isoproterenol, the NF-κB level was restored with no significant difference from the control (Figure 7).

### 3.7. TNF-α Protein Expression

**Figure 8:**
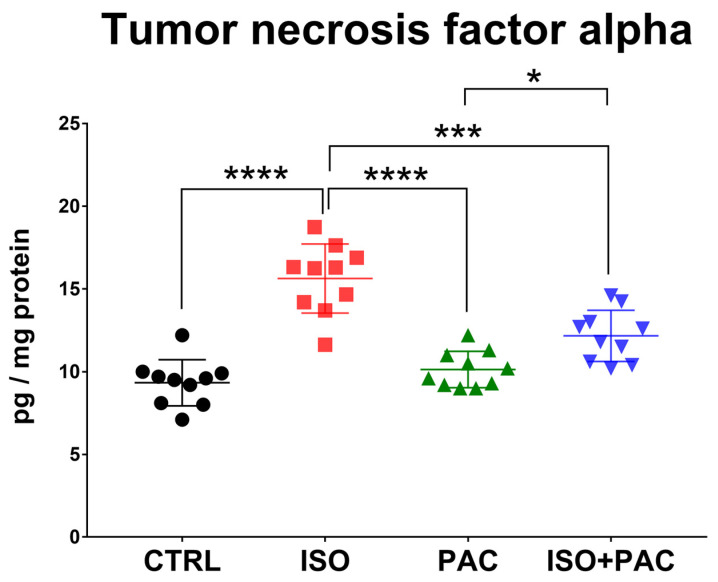
Tumor necrosis factor-α protein concentrations in CTRL-, ISO- and PAC-treated rat hearts. *: *p* < 0.05; ***: *p* < 0.001; ****: *p* < 0.0001.

TNF-α protein concentration was significantly increased in the isoproterenol-only group, while paclitaxel administration alone did not affect protein expression in control hearts. When paclitaxel was co-administered, the TNF-α level decreased compared to isoproterenol alone and was not significantly different from the control (Figure 8).

## 4. Discussion

The main finding of the current work is that paclitaxel protected the myocardium against isoproterenol-induced damage by decreasing inflammatory signals and increasing the concentration of antioxidant and anti-inflammatory molecules. Isoproterenol administration led to a significant elevation of the inflammatory molecules NF-κB, TNF-α and MPO, and the same time it depleted or inhibited the activity of the antioxidant and anti-inflammatory molecules HO-1, SOD and GSH in left ventricular tissue. On the other hand, when administered along with isoproterenol, paclitaxel restored the level of these molecules toward control levels.

This finding is in agreement with Cao and coworkers [14] who reported that taxol administration for 30 min during ligation-induced ischemia led to a significant increase in HO-1 protein expression in the rat heart. The increased protein concentration was associated with reduced ROS levels and the maintenance of the activity of mitochondrial complexes I and III. HO-1 is a stress-induced protein, which plays a crucial role in heme metabolism by catalyzing its conversion to free iron, carbon monoxide (CO) and biliverdin [20]. Biliverdin/bilirubin and CO possess anti-inflammatory, anti-apoptotic and antioxidant properties; thereby, the restoration of HO-1 protein expression in our study could contribute to the resistance against myocardial damage. On the other hand, in cultured cardiomyocytes, paclitaxel alone or in combination with angiotensin II induced apoptosis as indicated by an increased caspase-3 activity and terminal deoxy-nucleotidyl transferase-mediated dUTP nick end-labelling (TUNEL) assay [21]. These dissimilar conclusions may be explained by the different models used in these experiments. Moreover, it appears that paclitaxel in high doses may be detrimental, because similarly to our investigation, studies which reported a protective effect in the cardiovascular system employed a lower paclitaxel dose (up to 1 M vs. 1–10 μM). In fact, Kang et al. [17] demonstrated that doses over 1 μM increased inflammatory and decreased anti-inflammatory cytokine levels in smooth muscle cell-derived foam cells.

Similarly to HO-1, when paclitaxel was co-administered, SOD concentrations and total glutathione were also significantly different from the suppressed level in the isoproterenol group, although they did not completely return to control values in the current study. It is feasible that this effect of paclitaxel was realized via HO-1 expression, as Eltobshy and colleagues [22] also demonstrated in a myocardial injury model that chemical induction of HO-1 resulted in increased GSH and SOD activities compared to the isoproterenol-induced decrease. GSH functions as a direct antioxidant, helps recycle other antioxidants and protects against inflammatory responses [23], so when it is depleted, the cellular redox environment shifts to a prooxidant state. SOD is an enzymatic protective molecule which directly converts superoxide radicals and hydrogen peroxide to less reactive species [24]. It is likely that the antioxidant function of paclitaxel is manifested through the activation of the transcription factor nuclear factor erythroid 2-related factor 2 (Nrf2), which regulates the expression of all measured protective molecules; HO-1, GSH and SOD [25] also modulate the redox state of GSH via the regulation of glutathione reductase (GSR) [23].

Besides stimulating antioxidant and anti-inflammatory protein expressions, paclitaxel treatment also suppressed inflammatory molecules in the rat heart. Other investigations have previously demonstrated that isoproterenol exerts its myotoxic effects by promoting oxidative stress and inflammation [18,26]. MPO is considered an inflammatory marker whose activity increases with doxorubicin administration and pre-treatment with antioxidant compounds can effectively counteract this rise [27]. MPO also predicted coronary artery disease (CAD) and cardiovascular mortality risk in patients undergoing coronary angiography [28]. MPO, TNF-α and NF-κB all decreased when paclitaxel was used together with isoproterenol in our model; however, we did not measure the levels of ROS in the current study. Therefore, we cannot state with confidence whether this effect was reached through the suppression of oxidant production or the increased scavenging of ROS. Interestingly, Cao et al. [14] demonstrated that taxol infusion during cardiac ischemia maintained mitochondrial complex I activity, which is implicated in protection against ROS and cellular death [29].

**Figure 9:**
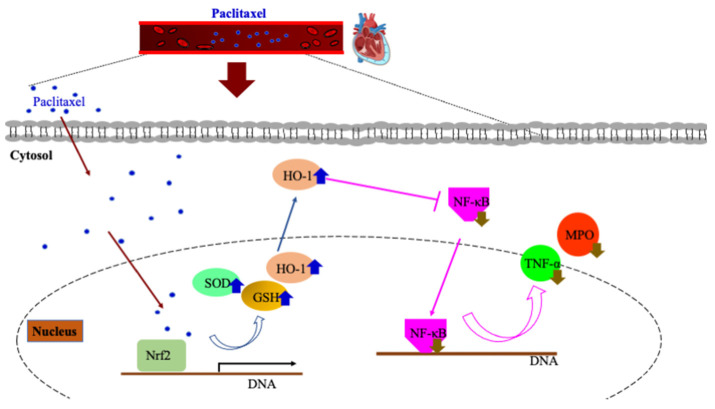
Potential cardioprotective mechanisms of paclitaxel.

Increased HO-1 expression is a potential mechanism that contributed to these effects (Figure 9). In fact, it was shown in diabetic mice that along with myocardial superoxide levels, plasma TNF-α was also reduced in response to chemically induced HO-1 elevation [30]. Since TNF-α is a major activator of NF-κB, the decreased expression in our study likely resulted in the suppression of this canonical proinflammatory signaling pathway [31]. In contrast to our findings, paclitaxel administration increased myocardial TNF-α concentration and apoptosis in mice as discussed in [16]. In this study, paclitaxel was administered every three days for three months via intraperitoneal injection, while our protocol included a 10 mg/kg paclitaxel dose for five days in an oral formulation by gavage [19]. Therefore, the exact dose, duration and route of administration appear to be critical for paclitaxel treatment, as they determine tissue exposure to the drug. In fact, it was stated that drug toxicity develops if high paclitaxel levels are maintained for an extended duration beyond five hours [19,32]. Importantly, paclitaxel dosing of 25 μg vs. 125 μg on a coated stent determined the myocardial concentration of the drug, with an approximately 10-fold increase with the high-dose stent compared to the low-dose used [33]. As opposed to previous research [16,21], in the present work paclitaxel administration alone did not affect any of the measured molecules in the ventricles without isoproterenol-induced damage. Others also demonstrated that paclitaxel did not induce any myocardial damage in isolated rat hearts during normoxia [8]. It is feasible that the oral dosing employed in the current study can prevent local toxicity [19]. Limitations of the current study include the lack of measures of morphological indices of heart damage or microtubular structures. However, it is well established that isoproterenol triggers structural and functional changes in rat myocardium similar to what is found in humans [18,25].

## 5. Conclusions

In conclusion, the current work demonstrated that oral administration of paclitaxel can effectively maintain the expression of the important antioxidant and anti-inflammatory molecules HO-1, SOD and GSH, and suppress the production of TNF-α, MPO and NF-κB that are involved in oxidative and inflammatory processes during myocardial damage. In addition, the principal component of this cellular defense seems to be the expression of HO-1, with a potential to reduce ROS, TNF-α, and in turn, NF-κB signaling in the myocardium [14,30,31]. Future studies should also analyze the levels of other cytokines, such as the members of the interleukin family (e.g., IL-1), which have been demonstrated to play a role in various inflammatory conditions in the heart [34,35]. In fact, IL-1 blockade reduced doxorubicin-induced damage in the myocardium, which may suggest a potential target in the ISO model as well [35]. Moreover, it would also be useful to investigate the expression of the transcription factor Nrf2 in the same model to elucidate whether paclitaxel has a direct effect on the de novo synthesis of the antioxidant and anti-inflammatory molecules.

## Figures and Tables

**Table 1:** Values of the measured parameters with mean and standard deviation.

Mean ± SD	Control	ISO	PAC	ISO + PAC
MPO (µU/mg of protein)	16,511 ± 5944	27,697 ± 4979	16,967 ± 3453	17,260 ± 2979
HO-1activity (nmol bilirubin/h/mg protein)	0.431 ± 0.0629	0.035 ± 0.0259	0.36 ± 0.1346	0.242 ± 0.05391
HO-1 (pg/mg of protein)	491.1 ± 58.87	237.8 ± 42.52	467 ± 50.29	381.4 ± 79.82
Total Glutathione (nmol/mg of protein)	3.894 ± 0.5405	1.758 ± 0.5383	3.718 ± 0.7018	2.762 ± 0.7259
SOD (ng/mL)	141.9 ± 3.1	64.6 ± 4.624	132 ± 9.95	109.1 ± 14.96
TNFα (pg/mg protein)	9.33 ± 1.395	15.63 ± 2.089	10.13 ± 1.103	12.16 ± 1.542
NFκB (pg/mg of protein)	189.4 ± 83.26	411.8 ± 42.69	119.6 ± 29.52	185.7 ± 47.8

MPO: myeloperoxidase, HO-1: heme oxygenase-1, SOD: Superoxide dismutase, TNFα: Tumor necrosis factor alpha, NFκB: Nuclear factor kappa B.

## Data Availability

The data presented in this study are available on request from the corresponding author.

## References

[1] Baumgartner I., Schindewolf M. (2020). The paclitaxel story in cardiovascular medicine. Eur. Heart J..

[2] Alfonso F., Rivero F., Granada J.F. (2020). Safety of Paclitaxel-Coated Balloons in the Coronary Arteries. J. Am. Coll. Cardiol..

[3] Mills J.L., Conte M.S., Murad M.H. (2019). Critical review and evidence implications of paclitaxel drug-eluting balloons and stents in peripheral artery disease. J. Vasc. Surg..

[4] Thompson C.A., Huibregtse B., Poff B., Wilson G.J. (2009). Time Dependent Vascular and Myocardial Responses of a Second Generation, Small Vessel, Paclitaxel-Eluting Stent Platform. Catheter. Cardiovasc. Interv..

[5] Spargias K., Gyöngyösi M., Hemetsberger R., Posa A., Pavo N., Pavo I.J., Huber K., Petrasi Z., Petnehazy O., von Strandmann R.P. (2014). Valvuloplasty with a Paclitaxel-Eluting Balloon Prevents Restenosis in an Experimental Animal Model of Aortic Stenosis. J. Heart Valve Dis..

[6] Pósa A., Nyolczas N., Hemetsberger R., Pavo N., Petneházy Ö., Petrasi Z., Sangiorgi G., Gyongyosi M. (2010). Optimization of Drug-Eluting Balloon Use for Safety and Efficacy: Evaluation of the 2nd Generation Paclitaxel-Eluting DIOR-Balloon in Porcine Coronary Arteries. Catheter. Cardiovasc. Interv..

[7] Beckman J.A., White C.J. (2019). Paclitaxel-Coated Balloons and Eluting Stents: Is There a Mortality Risk in Patients with Peripheral Artery Disease?. Circulation.

[8] Rodríguez-Sinovas A., Abad E., Sánchez J.A., Sanz C.F., Inserte J., Ruiz-Meana M., Alburquerque-Béjar J.J., García-Dorado D. (2015). Microtubule stabilization with paclitaxel does not protect against infarction in isolated rat hearts. Exp. Physiol..

[9] Steiner S., Schmidt A., Zeller T., Tepe G., Thieme M., Maiwald L., Schröder H., Euringer W., Ulrich M., Brechtel K. (2020). COMPARE: Prospective, Randomized, Non-Inferiority Trial of High- vs. Low-Dose Paclitaxel Drug-Coated Balloons for Femoropopliteal Interventions. Eur. Heart J..

[10] Katsanos K., Spiliopoulos S., Kitrou P., Krokidis M., Karnabatidis D. (2018). Risk of Death Following Application of Paclitaxel-Coated Balloons and Stents in the Femoropopliteal Artery of the Leg: A Systematic Review and Meta-Analysis of Randomized Controlled Trials. J. Am. Heart Assoc..

[11] Zeller T., Micari A., Scheinert D., Baumgartner I., Bosiers M., Vermassen F.E., Banyai M., Shishehbor M.H., Wang H., Brodmann M. (2020). The IN.PACT DEEP Clinical Drug-Coated Balloon Trial: 5-Year Outcomes. JACC Cardiovasc. Interv..

[12] Freisinger E., Koeppe J., Gerss J., Goerlich D., Malyar N.M., Marschall U., Faldum A., Reinecke H. (2020). Mortality after use of paclitaxel-based devices in peripheral arteries: A real-world safety analysis. Eur. Heart J..

[13] Cao H.-M., Wang Q., You H.-Y., Li J., Yang Z.-Y. (2011). Stabilizing Microtubules Decreases Myocardial Ischaemia-Reperfusion Injury. J. Int. Med. Res..

[14] Cao H., Wang Y., Wang Q., Wang R., Guo S., Zhao X., Zhang Y., Tong D., Yang Z. (2016). Taxol Prevents Myocardial Ischemia-reperfusion Injury by Inducing JNK-Mediated HO-1 Expression. Pharm. Biol..

[15] Kumazawa A., Katoh H., Nonaka D., Watanabe T., Saotome M., Urushida T., Satoh H., Hayashi H. (2014). Microtubule Disorganization Affects the Mitochondrial Permeability Transition Pore in Cardiac Myocytes. Circ. J..

[16] Ren S., Huang T., Ou D., Feng L., Huang S., Zhou C., Ge L. (2022). Inhibition of TNF-α and JNK Signaling Pathway Can Reduce Paclitaxel-Induced Apoptosis of Mouse Cardiomyocytes. Appl. Bionics Biomech..

[17] Kang Y., Cai Y., Pan W. (2022). Rapamycin and Paclitaxel Affect Human Aortic Smooth Muscle Cells-Derived Foam Cells Viability and Proliferation. Braz. J. Cardiovasc. Surg..

[18] Althunibat O.Y., Abduh M.S., Abukhalil M.H., Aladaileh S.H., Hanieh H., Mahmoud A.M. (2022). Umbelliferone Prevents Isoproterenol-Induced Myocardial Injury by Upregulating Nrf2/HO-1 Signaling, and Attenuating Oxidative Stress, Inflammation, and Cell Death in Rats. Biomed. Pharmacother..

[19] Kim D.-W., Kwon J.-S., Kim Y.-G., Kim M.S., Lee G.-S., Youn T.-J., Cho M.-C. (2004). Novel Oral Formulation of Paclitaxel Inhibits Neointimal Hyperplasia in a Rat Carotid Artery Injury Model. Circulation.

[20] Ryter S.W., Alam J., Choi A.M.K. (2006). Heme Oxygenase-1/Carbon Monoxide: From Basic Science to Therapeutic Applications. Physiol. Rev..

[21] Saji K., Fukumoto Y., Suzuki J., Fukui S., Nawata J., Shimokawa H. (2007). Colchicine, a Microtubule Depolymerizing Agent, Inhibits Myocardial Apoptosis in Rats. Tohoku J. Exp. Med..

[22] Eltobshy S.A., Hussein A.M., Elmileegy A.A., Askar M.H., Khater Y., Metias E.F., Helal G.M. (2019). Effects of Heme Oxygenase-1 Upregulation on Isoproterenol-Induced Myocardial Infarction. Korean J. Physiol. Pharmacol..

[23] Harvey C.J., Thimmulappa R.K., Singh A., Blake D.J., Ling G., Wakabayashi N., Fujii J., Myers A., Biswal S. (2009). Nrf2-Regulated Glutathione Recycling Independent of Biosynthesis Is Critical for Cell Survival during Oxidative Stress. Free Radic. Biol. Med..

[24] Scandalios J.G. (2005). Oxidative stress: Molecular perception and transduction of signals triggering antioxidant gene defenses. Braz. J. Med. Biol. Res..

[25] Chen Q.M., Maltagliati A.J. (2018). Nrf2 at the Heart of Oxidative Stress and Cardiac Protection. Physiol. Genom..

[26] Abukhalil M.H., Hussein O.E., Aladaileh S.H., Althunibat O.Y., Al-Amarat W., Saghir S.A., Alfwuaires M.A., Algefare A.I., Alanazi K.M., Al-Swailmi F.K. (2021). Visnagin Prevents Isoproterenol-Induced Myocardial Injury by Attenuating Oxidative Stress and Inflammation and Upregulating Nrf2 Signaling in Rats. J. Biochem. Mol. Toxicol..

[27] Sandamali J.A.N., Hewawasam R.P., Jayatilaka K.A.P.W., Mudduwa L.K.B. (2021). *Cinnamomum zeylanicum* Blume (Ceylon cinnamon) Bark Extract Attenuates Doxorubicin Induced Cardiotoxicity in Wistar Rats. Saudi Pharm. J..

[28] Heslop C.L., Frohlich J.J., Hill J.S. (2010). Myeloperoxidase and C-Reactive Protein Have Combined Utility for Long-Term Prediction of Cardiovascular Mortality after Coronary Angiography. J. Am. Coll. Cardiol..

[29] Sharma L.K., Lu J., Bai Y. (2009). Mitochondrial Respiratory Complex I: Structure, Function and Implication in Human Diseases. Curr. Med. Chem..

[30] Issan Y., Kornowski R., Aravot D., Shainberg A., Laniado-Schwartzman M., Sodhi K., Abraham N.G., Hochhauser E. (2014). Heme Oxygenase-1 Induction Improves Cardiac Function following Myocardial Ischemia by Reducing Oxidative Stress. PLoS ONE.

[31] Lawrence T. (2009). The Nuclear Factor NF-kappa B Pathway in Inflammation. Cold Spring Harb. Perspect. Biol..

[32] Gianni L., Kearns C.M., Giani A., Capri G., Viganó L., Lacatelli A., Bonadonna G., Egorin M.J. (1995). Nonlinear pharmacokinetics and metabolism of paclitaxel and its pharmacokinetic/pharmacodynamic relationships in humans. J. Clin. Oncol..

[33] Radeleff B., Lopez-Benitez R., Stampfl U., Stampfl S., Sommer C., Thierjung H., Berger I., Kauffmann G., Richter G.M. (2010). Paclitaxel-induced arterial wall toxicity and inflammation: Tissue uptake in various dose densities in a Minipig Model. J. Vasc. Interv. Radiol..

[34] Bartekova M., Radosinska J., Jelemensky M., Dhalla N.S. (2018). Role of cytokines and inflammation in heart function during health and disease. Heart Fail. Rev..

[35] Szekely Y., Arbel Y. (2018). A Review of Interleukin-1 in Heart Disease: Where Do We Stand Today?. Cardiol. Ther..

